# Systemic siRNA Delivery via Peptide-Tagged Polymeric Nanoparticles, Targeting PLK1 Gene in a Mouse Xenograft Model of Colorectal Cancer

**DOI:** 10.1155/2013/252531

**Published:** 2013-09-15

**Authors:** Meenakshi Malhotra, Catherine Tomaro-Duchesneau, Shyamali Saha, Satya Prakash

**Affiliations:** ^1^Biomedical Technology and Cell Therapy Research Laboratory, Departments of Biomedical Engineering, Faculty of Medicine, McGill University, 3775 University Street, Room 311, Lyman Duff Medical Building, Montreal, QC, Canada H3A 2B4; ^2^Faculty of Dentistry, McGill University, 3775 University Street, Montreal, QC, Canada H3A 2B2

## Abstract

Polymeric nanoparticles were developed from a series of chemical reactions using chitosan, polyethylene glycol, and a cell-targeting peptide (CP15). The nanoparticles were complexed with PLK1-siRNA. The optimal siRNA loading was achieved at an N : P ratio of 129.2 yielding a nanoparticle size of >200 nm. These nanoparticles were delivered intraperitoneally and tested for efficient delivery, cytotoxicity, and biodistribution in a mouse xenograft model of colorectal cancer. Both unmodified and modified chitosan nanoparticles showed enhanced accumulation at the tumor site. However, the modified chitosan nanoparticles showed considerably, less distribution in other organs. The relative gene expression as evaluated showed efficient delivery of PLK1-siRNA (0.5 mg/kg) with 50.7 ± 19.5% knockdown (*P* = 0.031) of PLK1 gene. The *in vivo* data reveals no systemic toxicity in the animals, when tested for systemic inflammation and liver toxicity. These results indicate a potential of using peptide-tagged nanoparticles for systemic delivery of siRNA at the targeted tumor site.

## 1. Introduction

Cancer is characterized by the uncontrolled growth of a group of cells that infest adjacent tissues and often metastasize to other organs via the lymphatic or circulatory system. It is primarily caused by environmental factors (90–95%), but also by genetic factors (5–10%) [1]. Typically the alteration in cell growth promoting oncogenes and cell division inhibiting tumor suppressive genes leads to the formation of cancer cells [2]. Depending on the stage of the cancer, the treatment options available include surgical removal, chemotherapy with anticancer drugs, such as 5-fluorouracil, oxaliplatin, and leucovorin [3], radiation therapy, immunotherapy [4], and hormone therapy with drugs like cetuximab and panitumumab [5]. However, it has been shown that cancers with genetic origin do not benefit from these chemotherapies [5]. Moreover, the toxicity and side-effects have severely limited the safety and effectiveness of these methods.

One of the target proteins in cancer therapy is serine/threonine-protein kinase (PLK1), a key regulator of mitosis in mammalian cells. PLK1 is a protooncogene overexpressed in a variety of human cancers [6, 7]. It is directly associated with p53, a tumor suppressor protein, and on interaction with p53, it inhibits the latter's transactivation and proapoptotic activity [8], leading to uncontrolled cell proliferation. Recently, the inhibition of PLK1 with antibodies, antisense oligonucleotides (ASOs), small interfering RNA (siRNA), or dominant negative mutants that suppress tumor growth by causing increased apoptosis have gained much interest as therapeutic options to treat cancer [9–14]. Although antineoplastic drugs have shown great success as a treatment for cancer therapy, many carcinomas are resistant to these agents, and thus, chemotherapy with these agents has become a major restriction at an advanced cancer stage [15, 16]. Some of the kinase inhibitors currently being investigated in clinical trials are BI2536 (phase II; Boehringer Ingelheim Pharmaceuticals), BI6727 (phase II; Boehringer Ingelheim Pharmaceuticals), GSK461364 (phase I; GlaxoSmithKline), NMS-1286937 (phase I; Nerviano Medical Sciences), and TAK-960 (phase I; Millennium Pharmaceuticals, Inc.) [17]. Another clinical trial is being conducted by Tekmira pharmaceuticals (TKM-080301), wherein the formulation comprises of a stable nucleic acid lipid particle containing siRNA against PLK1 targeted towards late-stage solid tumors [18]. Thus, siRNAs targeted against proliferation-associated signal transduction pathways, which can halt the tumor progression in animal models are emerging as an appealing approach. 

The delivery of siRNAs *in vivo* has been challenging for antitumor therapy due to their instability in physiological conditions, improper cellular distribution, low bioactivity, repeated dosage requirement, and the necessity for continuous long-term infusions [19]. Various commercially available delivery/transfection reagents can provide better siRNA delivery in substantially lower doses than siRNA delivered alone, but these have concerns of target specificity, cytotoxicity, immunological responses, stable systemic delivery, and off-target effects of these reagents [20, 21]. Moreover, the efficacy of most of these commercially available transfection reagents is limited to *in vitro* use [22–25]. For *in vivo* applications, delivery via a systemic route targets multiple sites, which may not be an ideal deal for many biomedical applications. Thus, the development of a delivery vehicle that can overcome these issues and identify cell-specific receptors, expressed as tumor biomarkers, with an ability to distinguish between cancer and normal cells, will be an effective approach to overcome the limitations of currently used therapeutics [26, 27]. 

The current study proposes a peptide-tagged polyethylene glycol (PEG)ylated chitosan nanoparticle for *in vivo* siRNA delivery. The target specificity of the nanoparticles is attributed to a peptide that guides the nanoparticle system carrying siRNA to specific tissues, when administered via a systemic route. In this study, we have used a peptide, CP15, on the nanoparticles, which has been identified by the generation of phage displayed libraries [28]. The method of phage display library for identifying specific binding ligands has found wide application in isolating peptides that have high binding affinity for cancer cells [28]. The advantage of using peptide-based tumor targeting is their rapid clearance from the blood because of their small size and lack of immunogenicity. CP15 peptide has shown to be the most effective peptide targeting colon tumor cells, while not recognizing normal human intestinal epithelial cells [28]. The nanoparticle formulation developed in this study was used to selectively target the tumor tissue, expressing affinity towards CP15 peptide, in a mouse xenograft model of colon cancer developed from SW480 epithelial colon cancer cells. This current study illustrates an example and potential use for the siRNA/nanoparticle formulation in cancer therapy.

## 2. Methods

### 2.1. Materials

Chitosan at a molecular weight of 50 kDa–190 kDa, with viscosity 20–300 cP and degree of deacetylation of 75–85%, polyethylene glycol monomethyl ether (mPEG) (M.W. 2,000), and sodium tripolyphosphate (TPP) and glacial acetic acid of analytical grade were obtained from Sigma (Oakville, ON, Canada). CP15 (VHLGYAT-NH_2_), M.W. 758.3, was synthesized by Sheldon Biotech, McGill University. Biotin-tagged scrambled siRNA, siGENOME non-targeting siRNA number 2: D-001210-02, and PLK1 siRNA (h) with sequence; PLK1 (sense strand)—5′AGAmUCACCCmUCCUmUAAAmUAUU 3′—and PLK1 (antisense strand)—5′UAUUUAAmGGAGGGUGAmUCUUU 3′—were procured from Dharmacon (Lafayette, CO, USA), where “m” represents 2′ O methylated nucleotide. 

### 2.2. Preparation of siRNA-Nanoparticle Formulation

The nanoparticles were prepared from the chemically modified polymer (CS-PEG-CP15), which was synthesized using a well-established protocol as previously published by our group [29]. For nanoparticle preparation, the derivatized polymer CS-PEG-CP15 (0.5 mg/mL) was dissolved in 1% acetic acid solution (pH 5.0). The polymer was heated at 60°C and sonicated to ensure maximum dissolution. The polymer was filtered using 0.8 *μ*m filter before forming nanoparticles. TPP at 0.7 mg/mL, pH 3.0, was used as a crosslinker to form nanoparticles. The biotin-tagged scrambled siRNA or PLK1 siRNA, in required amounts, as calculated for varied nitrogen : phosphorous (N : P) ratios (51.6, 75, 103, 129.2, and 155), was premixed with 200 *μ*L of TPP and dropped into the 800 *μ*L of CS-PEG-CP15 polymer solution, under constant magnetic stirring at 800 rpm for an hour. The N : P ratio (molar ratio of chitosan amino groups/siRNA phosphate groups) was calculated using 325 Da as mass-per-phosphate and 161.16 (molecular weight of repeating units of chitosan) as mass-per-charge for chitosan.

### 2.3. Gel Retardation Assay

The loading efficiency of siRNA with derivatized polymer, CS-PEG-CP15, at varying N : P ratios was determined by gel electrophoresis on a 4% (w/v) agarose gel. Varying N : P ratios, 51.6, 75, 103, 129.2, and 155, along with 1 : 6 dilution of the 6x orange dye was loaded onto the gel and was run for 4 hours at 55 V in Tris-borate EDTA (TBE) buffer (pH 8.3). The TBE buffer contained ethidium bromide at a concentration of 0.5 *μ*g/mL, which was required for the visualization of RNA bands under UV transillumination at 365 nm.

### 2.4. Characterization of siRNA-Nanoparticle Formulation

The nanoparticles prepared at varying N : P (chitosan : siRNA) ratio were analyzed morphologically for their relative size under transmission electron microscopy (TEM) JEOL JEM-2000fx transmission electron microscope (Tokyo, Japan). The nanoparticle size and zeta potential of the chitosan nanoparticles, PEGylated chitosan nanoparticles, and peptide-tagged PEGylated chitosan nanoparticles were analyzed using Brookhaven BI-90 Particle Nanosizer (Holtsville, NY, USA).

### 2.5. Animals and *In Vivo* Tumor Induction

Six week old Balb/c nude mice, weighing 15–20 g were purchased from Charles River Laboratories (Wilmington, MA, USA) and housed in an environment with controlled temperature (22°C), humidity, and a 12 h light/dark cycle at McGill's Animal care facility. The animal experiment was conducted as per the protocol approved by the Animal Care Committee at McGill University (Montreal, QC, Canada). Standard mouse chow pellets and water were supplied ad libitum. For tumor induction, animals were subcutaneously injected with 100 *μ*L of SW480 colon cancer cells (2 × 10^6^) mixed with an equal volume of matrigel (BD). The treatment began after the tumor reached a volume of 100 mm^3^. The tumor size was measured using a digital caliper and was calculated by the formula volume = (width)^2^ ×  length/2. 

### 2.6. Animal Study

Animals were acclimatized for a week before the start of the experiment. The animals were randomized into 4 treatment groups (*n* = 6) to receive treatment formulations: (1) CS-PEG-CP15 with PLK1 siRNA, (2) CS-PEG-CP15 with scrambled siRNA, (3) PLK1 siRNA alone, and (4) 0.85% NaCl as a control. In each treatment group, the animals received a total siRNA dose of 0.5 mg/kg animal mass. The dose 0.5 mg/kg of siRNA has been optimized as an optimal *in vivo* dose delivery in our previous study (data not presented). 100 *μ*L of treatment formulations were administered every alternate day via intraperitoneal injections for a period of 2 weeks. 

### 2.7. Biodistribution Study to Identify siRNA Delivery by Nanoparticles in Various Organs

Biodistribution study was performed separately on an animal from each group, receiving different formulations as (a) CS-PEG-CP15 with scrambled biotin-siRNA, (b) chitosan with scrambled biotin-siRNA, (c) scrambled biotin-siRNA, and (d) 0.85% NaCl as control. The animals were sacrificed after 4 h of intraperitoneal dose administration. Histopathological analysis was performed on tumor, lungs, heart, kidney, spleen, and liver. In brief, the tissues were harvested and kept at 4°C in 10% phosphate buffered formalin for 48 hours. The tissues were then trimmed to 3 mm thick sections and stored at 70% ethanol in histology cassettes. The tissues were paraffin embedded and processed into 4 *μ*m thick section on slides at the histology core facility (The Rosalind and Morris Goodman Cancer Research Centre, McGill University). The tissue section on slides were stained with Vectastain elite ABC kit (Vector laboratories; Burlingame, CA, USA) as per the manufacturer's protocol, and diaminobenzidine (DAB) was used as a substrate to assess the presence of biotin, used as a tag on siRNA for histology identification purposes. Haematoxylin was used as a counterstain, and slides were mounted with permount (Vector laboratories; Burlingame, CA, USA) and observed under compound microscope (Leica DM500; Ontario, Canada) at 400x.

### 2.8. Relative PLK1 Gene Expression by qPCR Analysis

After 2 weeks of treatment, the animals were sacrificed and the tumor tissues were harvested. A quantitative real-time PCR was performed (*n* = 6) to evaluate the percentage endogenous PLK1 gene expression. The total RNA was extracted using RN*easy* Lipid tissue mini kit from Qiagen, and the total RNA was quantified using Nanodrop 2000 spectrophotometer. The reverse transcription on total RNA was performed to obtain complementary DNA (cDNA) using a QuantiTect Reverse Transcription kit from Qiagen. A quantitative real-time PCR was performed using MBI Evolution Evagreen Master Mix following the manufacturer's protocol (MBI, Montreal, Canada) on ECO RT PCR machine from Illumina. The relative expression levels of PLK1 gene were normalized with the housekeeping gene GAPDH. The primer sequences used were as follows: PLK1, 5′-GGCAACCTTTTCCTGAATGA-3′ and 5′-AATGGACCACACATCCACCT-3′; GAPDH, 5′-TAAAGGGCATCCTGGGCTACACT-3′ and 5′-TTACTCCTTGGAGGCCATGTAGG-3′. The PCR was run for 30 to 40 cycles with a 95°C denaturing step (5 s), a 60°C annealing step (15 s), and a 72°C extension step (15 s), plus final incubation at 72°C for 10 min.

### 2.9. Protein Extraction and Western Blot Analysis

The harvested tumor tissues were preserved in “All protect tissue reagent” from Qiagen (Toronto, ON, Canada). The tissue samples were homogenized using a PowerGen Model 125 Homogenizer from Fisher Scientific (Ottawa, Ontario, Canada) at 26,300 rpm in 2 mL of ice cold RIPA buffer (20 mM Tris pH 8, 150 mM NaCl, 5 mM EDTA, 1% Nonidet P-40, 0.1% SDS, 10.0% glycerol, 10 mM Na_2_HPO_4_·7H_2_O, 1% sodium deoxycholate) containing phenylmethylsulfonyl fluoride (PMSF) and protease inhibitor cocktail from Roche Diagnostics (Laval, QC, Canada). The crude extract was incubated on ice for 30 minutes and centrifuged at 10,000 ×g for 10 minutes at 4°C to remove tissue debris. The supernatant was collected, and the protein concentration was determined using Pierce BCA Protein assay kit from Thermo Scientific (Rockford, IL, USA). Briefly, aliquots containing 100 *μ*g of protein were heated at 70°C for 15 minutes with NuPAGE LDS sample buffer supplemented with 100 mM DTT. The proteins were fractionated on precast NuPAGE 4–12% Bis Tris Gel from Invitrogen (Ontario, Canada) at 200 V for 35 minutes in MES SDS running buffer. Magic mark 1 Kb protein ladder was used as a standard. The gel was electrophoretically transferred to a 0.45 *μ*m pore size Novex nitrocellulose membrane using NuPAGE transfer buffer on a Novex SemiDry blotter (Invitrogen, ON, Canada). After transfer, the nitrocellulose membrane was incubated for 1 hr in 5% nonfat powdered milk in 1x Tris buffered saline (TBS) buffer supplemented with 0.2% Tween 20. The membrane was then incubated overnight at 4°C with mouse PLK (F-8) monoclonal antibody (1 : 100 dilution). The next day, the membrane was washed thrice with TBST for 15 minutes each and then incubated with HRP-conjugated goat antimouse IgG secondary antibody (1 : 2,000 dilution). The membrane was again washed thrice with TBST followed by the detection of the signal with chemiluminescent agents (ECL, Amersham) from GE healthcare. The bound antibody was visualized using autoluminography. To control the protein loading, the membrane was reprobed with primary mouse monoclonal *β*-actin (C-4) antibody (1 : 1000) with an overnight incubation at 4°C, followed by three washes in TBST and detection with HRP-conjugated goat anti-mouse IgG secondary antibody (1 : 2,000) and development with chemiluminescent agents, as described earlier. The protein bands developed using autoluminscence were quantified using Image J software (NIH, USA) and plotted with animal numbers, *n* = 6 in each group.

### 2.10. Serum Collection and Analysis 

Serum was collected via cardiac puncture using a sterile 23 G/25 mm needle. Approximately 400 *μ*L of blood was collected in the Microtainer serum separator tubes (Becton Dickinson, NJ, USA). The blood was allowed to clot at room temperature for 30 minutes and subsequently placed on ice until centrifugation. Serum was separated by low-speed centrifugation at 3600 rpm for 8 min at 4°C. The separated serum was frozen at −80°C until analysis. Serum was used to test C-reactive protein (CRP) and liver function tests, alanine aminotransferase (ALT), aspartate transaminase (AST), using a conventional enzymatic method on Hitachi 911 automated clinical chemistry autoanalyzer (Roche Diagnostics, USA).

### 2.11. Statistical Analysis

Experimental results are expressed as scattered dot plots with median. Statistical analysis was carried out using SPSS Version 17.0 (Statistical Product and Service Solutions, IBM Corporation, New York, NY, USA). Statistical comparisons were carried out using the General Linear Model and Tukey's post hoc analysis. Statistical significance was set at *P < 0.05, and **P < 0.01 were considered highly significant.

## 3. Results 

### 3.1. Synthesis and Characterization of CP15-Tagged PEGylated Chitosan Nanoparticles

**Figure 1:**
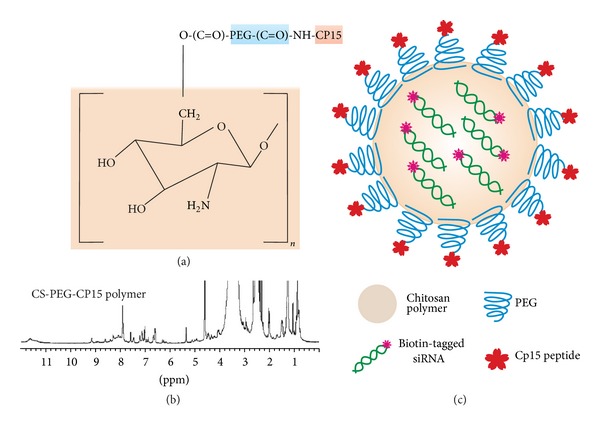
(a) Chemical structure of chitosan-PEG-CP15 polymer; (b) ^1^H NMR spectra of chitosan-PEG-CP15 (CS-O-PEG-CONH-CP15). The multiple peaks of oxymethyl groups in PEG at *δ* 3.3 to 3.7 cover over the signals of pyranose ring of chitosan. The multiple peaks at *δ* 6.0–9.0 belong to the peptide CP15 sequence, respectively. (c) A nanoparticle formulation with a core formed by chitosan polymer, surface functionalized with PEG, and a cell-targeting peptide (CP15). The formulation encases a biotin-tagged siRNA.

**Figure 2:**
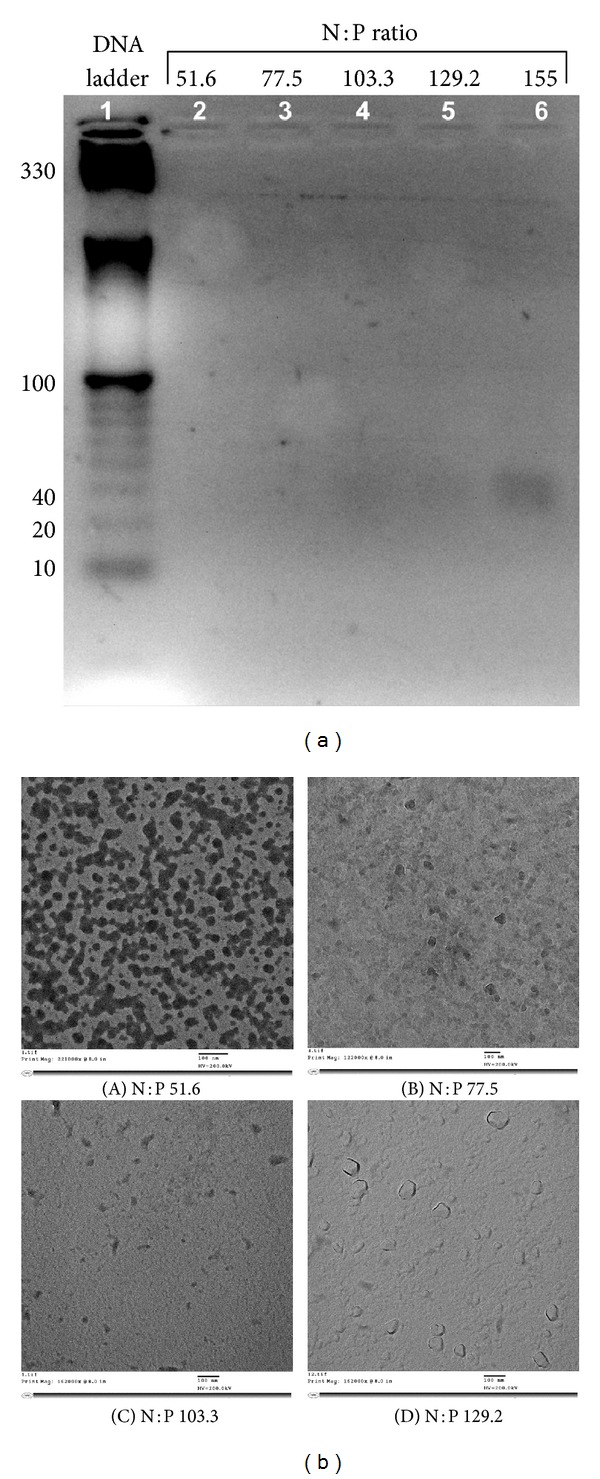
(a) Gel retardation assay was performed to evaluate maximum gene (siRNA) loading efficiency in the nanoparticles. Lane 1 represents a 10 bp DNA ladder used as a reference. Lanes 2, 3, 4, 5, and 6 represent various N : P ratios tested. The N : P ratio of 129.2 showed optimal siRNA loading with CS-PEG-CP15 polymer solution. (b): TEM image of CS-PEG-CP15/siRNA nanoparticles complexing siRNA at various N : P ratios: (A) 51.6 (mag. 221000x), (B) 77.5 (mag. 122000x), (C) 103.3 (mag. 162000x), and (D) 129.2 (mag. 95800x).

The peptide-tagged PEGylated chitosan polymer (CS-PEG-CP15) was synthesized following a series of chemical reactions, as previously published by our group [29]. Figures 1(a) and 1(b) represent a chemical structure and the NMR spectra, respectively, of the synthesized polymer, wherein the multiple peaks of the oxymethyl groups in PEG at *δ* 3.3 to 3.7 cover the signals of the pyranose ring of chitosan in the spectra. The weak and broad peak at *δ* 4.3–4.5 is from the protons of –NH–CH(CH_2_)–CO– in CP15 peptide. The multiple peaks at *δ* 6.0–9.0 belong to the CP15 peptide sequence. Nanoparticles were prepared following an ionic gelation scheme, as described previously by our group [30], wherein the cationic polymer complexes the anionic molecule (siRNA) due to the electrostatic interaction.Figure 1(c) shows a schematic illustration of the nanoparticle-siRNA formulation. The siRNA-nanoparticle formulations were prepared at varying N : P ratio and the optimal siRNA loading efficiency was determined by the gel retardation assay (Figure 2(a)). Results indicate that at an N : P ratio of 129.2, siRNA was completely complexed by the CP15-tagged PEGylated chitosan polymer, to form nanoparticles. The total siRNA that was complexed by the nanoparticles was calculated to be 8 *μ*g/mL. The siRNA-nanoparticle formulations prepared at different N : P ratios were observed under TEM (Figure 2(b)). The hydrodynamic size, measured by dynamic light scattering (DLS) and zeta potential (*ζ*) measurements of the three different nanoparticle formulations prepared at an N : P ratio of 129.2, were as follows: (a) chitosan nanoparticles; size: 212.1 ± 5.4 nm and *ζ*: 39.57 ± 1.37 mV, (b) PEGylated chitosan nanoparticles; size: 231.6 ± 8.2 nm and *ζ*: −5.44 ± 2.49 mV, and (c) peptide-tagged PEGylated chitosan nanoparticles; size 263.6 ± 13.5 nm and *ζ*: −7.59 ± 0.94 mV. 

### 3.2. Tumor Accumulation of siRNA-Nanoparticle Formulation

**Figure 3:**
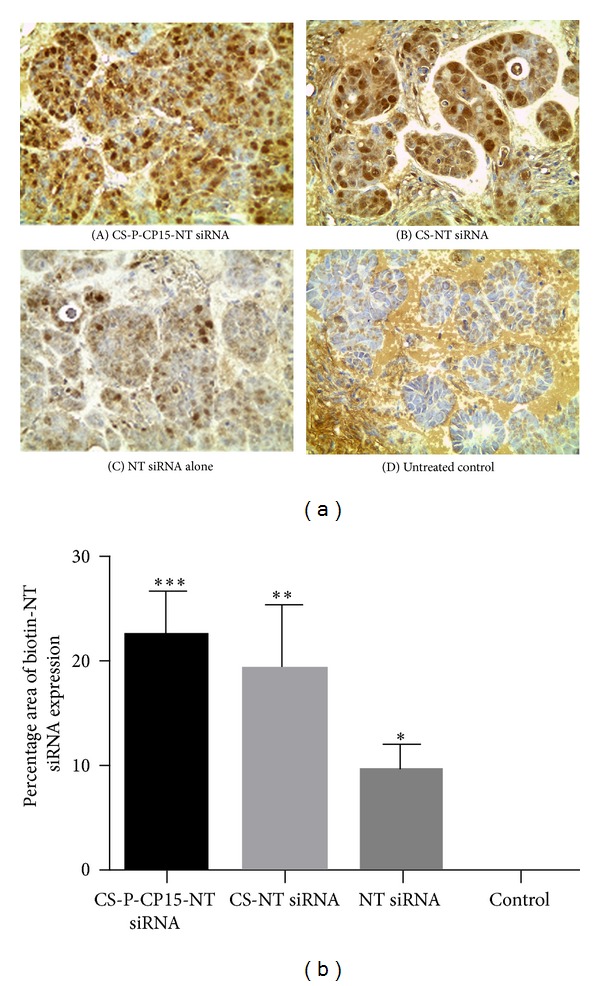
Analysis of nanoparticles accumulation in the tumor tissue after 4 h of dose administration. (a) Histopathological images of SW480 colon cancer tissue stained dark brown in color, indicating the presence of scrambled biotinylated siRNA (0.5 mg/kg) in the tumor tissue after intraperitoneal administration of the treatment nanoparticle formulation. Animals were sacrificed after 4 hrs. (A) Chitosan-PEG-CP15 (B) Unmodified chitosan nanoparticles (C) non-targeting biotin siRNA alone, (D) control: untreated. (b) Image analysis of the mean percent area stained in the tumor tissues. The graph shows a representative result of the average of three random sections measured per animal tissue, mean ± SD. *P < 0.05, **P < 0.01.

 The optimized siRNA-nanoparticle formulation prepared at N : P ratio of 129.2 was used for *in vivo* studies. The biotin tag facilitated the identification of siRNA in the tumor tissues, providing dark brown cellular stains. As represented in Figure 3(a), the histopathological images of the tumor tissues, stained dark brown in color, indicate the presence of biotin-tagged siRNA. The staining was found to be approximately equal and significant for both (a) CS-PEG-CP15-siRNA-nanoparticle formulation (*P* = 0.00043) and (b) unmodified chitosan-siRNA-nanoparticle formulation (*P* = 0.0011) in comparison to the untreated control group. The staining of (c) scrambled biotin-siRNA, delivered alone, was comparatively low in the tumor tissues when compared with the other treatment groups and was not significant, when compared to the untreated control (*P* = 0.062).  Figure 3(b) represents the mean percentage area analyzed for intensity, in triplicates from an animal tissue in each treatment, using Image J software. 

### 3.3. Biodistribution of siRNA-Nanoparticle Formulation to Other Organs

**Figure 4:**
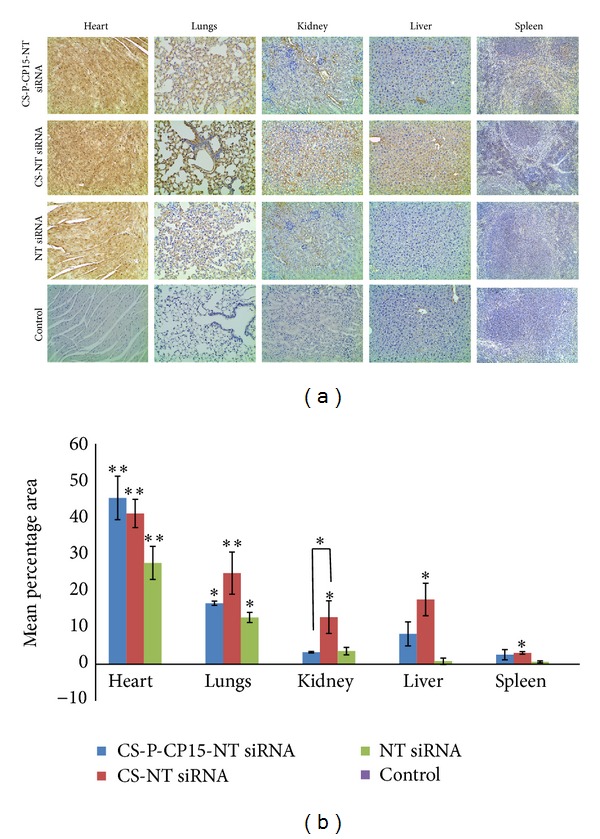
Analysis of biodistribution of siRNA in various tissues after 4 hours of dose administration. (a) Histopathological images of heart, lungs, kidney, liver, and spleen obtained from a mouse xenograft model of colon cancer. The represented dark brown staining in the tissues emphasizes the presence of scrambled biotinylated siRNA (0.5 mg/kg) with different treatment formulations: chitosan-PEG-CP15, Unmodified chitosan nanoparticles, non-targeting biotin-siRNA alone, and control as untreated. (b) Image analysis of the mean percent area stained in the tumor tissues. The graph shows a representative result of the average of two random sections measured per animal tissue, mean ± SD. *P < 0.05, **P < 0.01.

The biodistribution analysis for the three different treatments (a) CS-PEG-CP15-siRNA, (b) unmodified chitosan-siRNA, and (c) siRNA alone was evaluated in various other organs: heart, lungs, kidney, liver and spleen, and were compared with the untreated control (Figure 4(a)). The results indicate significant biodistribution of the unmodified chitosan nanoparticles in all the organs (*P* < 0.05) when compared with the control. For CS-PEG-CP15 with siRNA-nanoparticle formulation, the biodistribution was found to be significant only in the heart (*P* = 0.001) and lungs (*P* = 0.017), as compared with the control. Likewise, siRNA delivered alone showed significant staining in the heart (*P* = 0.009) and lungs (*P* = 0.043). Figure 4(b) represents the mean percentage area analyzed for intensity in duplicate from an animal tissue in each treatment, using Image J software.

### 3.4. QPCR Analysis of PLK1 Gene Expression in Tumor Tissues

**Figure 5:**
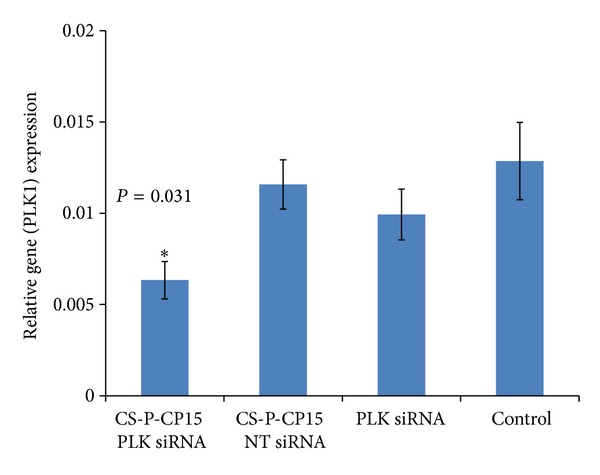
Quantitative PLK1 mRNA analysis in the tumor tissue. The graph represents relative gene expression of PLK1 in tumor tissues, after intraperitoneal administration of various treatment formulations; (i) CS-PEG-CP15 with siRNA against PLK1 gene, (ii) CS-PEG-CP15 with non-targeting biotin-siRNA, (iii) PLK1 siRNA alone, and (iv) control: saline. The PLK1 gene expression was compared among different groups after normalizing the GAPDH levels among all the animals. A 50.7 ± 19.5% PLK1 gene suppression was observed in treated animals (*P* = 0.031) as compared with untreated control. The graph shows a representative result of *n* = 6 in each group, mean ± SE. *P < 0.05 was considered significant based on Tukey's post hoc analysis, when compared with other groups.

The PLK1 gene expression was evaluated by extracting total RNA from the tumor tissues. The total RNA was reverse-transcribed to cDNA and was quantified by real-time PCR, using specific primers for the PLK1 gene. The percentage gene knockdown of PLK1 by siRNA delivered through CS-PEG-CP15 nanoparticles was evaluated by comparing the treatment group with other controls; mock transfections (CS-PEG-CP15 with scrambled biotin-siRNA), PLK1 siRNA alone, and 0.85% NaCl as control. As represented in Figure 5, the treatment group showed 50.7 ± 19.5% PLK1 gene knockdown (*P* = 0.031) when compared with the untreated control (*n* = 6). No significant difference was observed among groups that received mock transfections, that is, nanoparticles with scrambled biotin-siRNA (*P* = 0.933) and PLK1 siRNA alone (*P* = 0.539), when compared with the untreated control. This result confirmed that the nanoparticles were able to safeguard the siRNA, when administered systemically, and the siRNA retained its functional ability of gene knockdown and was not degraded by the serum proteins.

### 3.5. Analysis of PLK1 Protein Suppression by Western Blot

**Figure 6:**
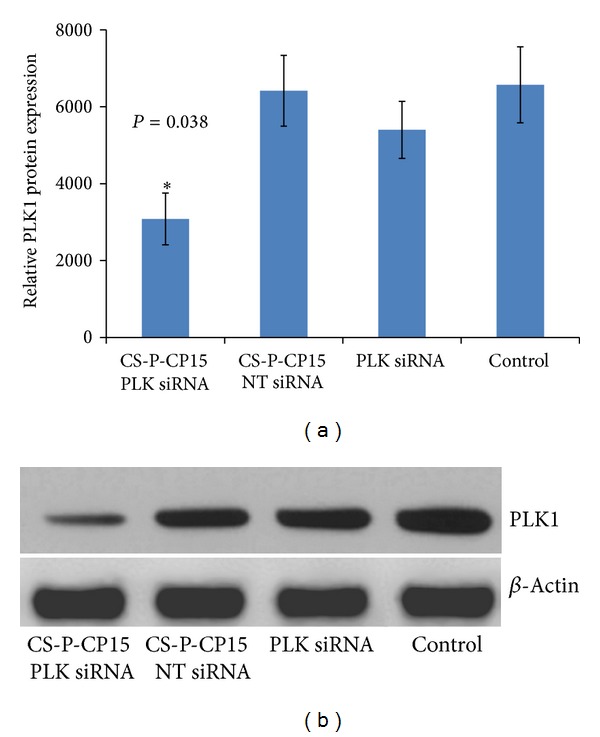
Western blot analysis from 100 *μ*g of total protein extracted from tumor tissues of colon cancer. The PLK1 protein expression was compared among different groups after normalizing the *β*-actin levels among all the animals. The graph (a) represents relative protein expression of PLK1 gene in tumor tissue after intraperitoneal administration of various treatment formulations; (1) CS-PEG-CP15 with siRNA against PLK1 gene, (2) CS-PEG-CP15 with non-targeting biotin-siRNA, (3) PLK1 siRNA alone, and (4) untreated control: saline. Reduction in PLK1 protein expression was observed with treatment formulation (1) as compared with untreated and mock transfection controls. The graph shows a representative result of *n* = 6 in each group, mean ± SE. *P < 0.05 was considered significant based on Tukey's post hoc analysis, when compared with other groups.

The relative protein expression, as observed in Figures 6(a) and 6(b), showed that the animals receiving the CS-PEG-CP15/siRNA (PLK1) nanoparticle formulation showed a significantly lower expression of PLK1 (*P* = 0.038), when compared with the untreated control. However, no difference was observed among the mock transfection groups, that is, nanoparticles with scrambled biotin-siRNA (*P* = 0.999) and PLK1 siRNA (*P* = 0.758) alone when compared with the untreated, control group. These results suggest that the siRNA targeted against PLK1 was solely responsible for the decrease in the protein expression at the tumor site and there were no deleterious effects posed by the polymeric nanoparticle formulation on the tumor.

### 3.6. Serum Analysis for Safety and Toxicity Study

**Figure 7:**
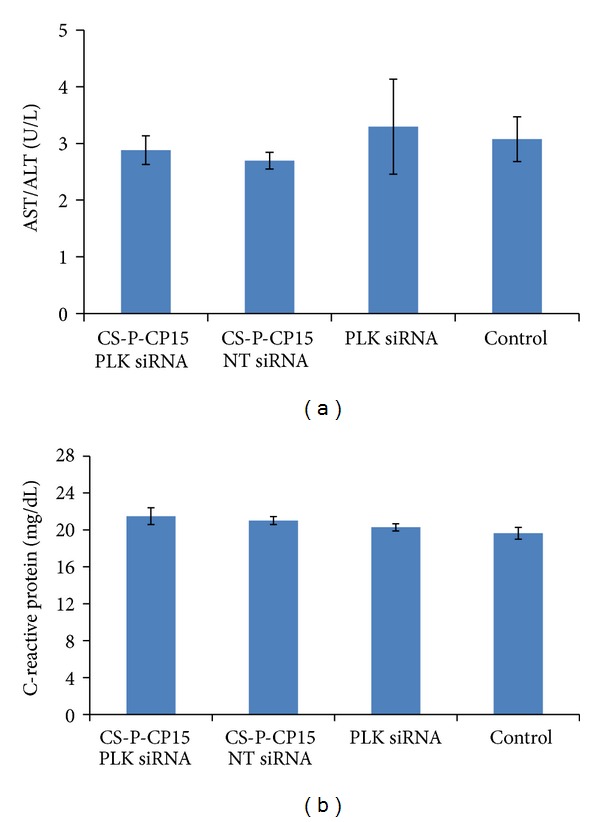
Serum analysis was performed as a safety test for comparing the (a) CRP and (b) ALT, AST levels among different treatment groups. The results indicate no significant difference between the treatment group (CS-PEG-CP15) containing siRNA against PLK1 gene with mock transfections and untreated controls. This concludes that the treatment formulation had no deleterious effect in terms of toxicity and immunological reactions on the animals. The graph shows a representative result of *n* = 5 in each group, mean ± SE, with no statistical significance according to Tuckey's post hoc analysis, when compared with other groups.

The serum was analyzed for safety tests, specifically for liver function tests AST/ALT and CRP as represented in Figures 7(a) and 7(b), respectively. No significant differences were observed among any treatment groups, when compared with the control (*n* = 5) for both AST/ALT and CRP. Thus, this study suggests that the synthesized CS-PEG-CP15-nanoparticle formulation did not cause any systemic toxicity to the animals.

## 4. Discussion

The advent of nanodelivery vehicles to facilitate the delivery and targeting of therapeutic molecules is becoming of great interest. The targeted delivery approach can increase the bioavailability of the drug at the tumor site. In addition, it encases the therapeutic payload, minimizing its toxic exposure to the surrounding tissue and in turn protecting it from degrading enzymes. The hyperproliferative environment of the tumor demands additional energy from its surroundings resulting in the generation of an acidic environment [31]. The disorganized and leaky endothelial junctions, with a size in the range of 100–780 nm, allow the uptake of nanovectors via passive targeting by a phenomenon called enhanced penetration and retention (EPR) effect [32]. However, active targeting can also take place by using ligands showing specific affinity towards a particular cell [29].

In this study, we utilized a derivatized chitosan nanoparticle, surface graphed with PEG and tagged with a CP15 peptide. The utility of chitosan as a parent polymer for the nanoparticle preparation was preferred due to its inherent “proton sponge character,” which enables endosomal escape of the nanoparticles [33]. This phenomenon is specifically important for nucleic acid delivery, such as siRNA in this study. After cellular uptake, the amines of chitosan nanoparticle get protonated after encountering the acidic pH of endosomal vesicle. This leads to endosomal swelling and lysis, thereby releasing the siRNA into the cytoplasm [33]. Keeping this phenomenon in mind, the derivatized chitosan polymer, synthesized for the nanoparticle preparation, was PEGylated at the C2 hydroxyl group of the chitosan polymer (Figure 1(a)). The detailed synthesis of the polymer is published elsewhere by our group [29]. Figure 1(b) represents the NMR spectra of the final CS-PEG-CP15 synthesized polymer, and Figure 1(c) represents the schematic of the nanoparticle after siRNA complexation, formed via ionic gelation method. These nanoparticles have been tested *in vitro* for their ability to deliver siRNA in mammalian cancerous cells and showed efficient siRNA delivery with minimal cytotoxicity as compared to the unmodified chitosan nanoparticles, published elsewhere [29].

The current study engages these nanoparticles to deliver siRNA against PLK1 gene and exhibit significant knockdown *in vivo*, in a mouse xenograft model of colorectal cancer. The results indicate complete complexation of siRNA with the derivatized chitosan polymer (CS-PEG-CP15) at N : P ratio of 129.2 (Figure 2(a)) with a hydrodynamic size of >200 nm as determined by dynamic light scattering (DLS). The ratios lower than 129.2 showed clumping of nanoparticles due to the presence of excess cationic polymer and lack of sufficient anionic siRNA in the solution to complex and form nanoparticles (Figure 2(b)), and at an N : P ratio of 155 the siRNA was apparently visible in the gel. Thus, the optimal determination of the N : P ratio was made collectively by considering the nanoparticle morphology under TEM and the gel retardation assay. Therefore, a ratio of 129.2 was considered optimal for the current study. Another similar study showed the chitosan/siRNA nanoparticles prepared at an N : P ratio of 150 led to 80% gene silencing of the endogenous enhanced green fluorescent protein (EGFP) in H1299 human lung carcinoma cells [34]. The efficiency of gene knockdown was related to the nanoparticles prepared at a high N : P ratio, which leads to the anticipation that at a high N : P ratio, the chitosan is loosely associated with the formation of nanoparticles and thus contributes to improved stability and gene silencing. The size of the nanoparticle depends on the molecular weight of the parent (chitosan) polymer, which was 50–190 kDa in this study. The nanoparticles developed with this molecular weight range were considered optimal for the current study to avoid glomerular filtration of the nanoparticles (size < 5 nm), when administered systemically. The chitosan nanoparticles as developed were observed to have increased in size with grafting of each PEG and further with attachment of the targeting peptide. However, the zeta potential was observed to decrease after PEGylation and attachment of the targeting peptide. This showed successful modification of chitosan polymer with successive moieties. The advantage of incorporating PEG also attributes to its properties of providing “stealth” character to the nanoparticles. PEG, being hydrophilic in nature, enhances the particle stability in dispersion and avoids plasma protein identification and escape from opsonization and clearance. Thus preventing uptake and clearance by reticuloendothelial system (RES) [35, 36].

Many studies have indicated the uptake of PEGylated nanoparticles in the range of 100–200 nm to be taken up by the tumor tissues [37, 38]. It has been observed that a greater influence of tumor uptake and prevention from RES is supported by the density of PEG on the nanoparticles [39, 40]. The PEG density has been classified into mushroom- or brush-like conformation, wherein it is observed that brush-like conformation has shown greater tumor uptake and longer circulation time [41]. Thus, molecular weight of PEG and its density on the nanoparticles play a very important role. The optimization of PEG on nanoparticles has been previously published by our group [42]. Though the use of PEG offers various advantages, it can lead to the generation of anti-PEG IgM antibodies on repeated administration, *in vivo*. The anti-PEG IgM produced in the spleen leads to clearance of the next dose of PEGylated nanoparticles from the system. This phenomenon has been termed as accelerated blood clearance (ABC), which was reported by Ishida and Kiwada [43]. Referring to this phenomenon, replacement of PEG with polymers like poly(N-vinyl-2-pyrrolidone) (PVP) has been utilized, which has shown improved blood circulation without ABC phenomenon [44]. Another recent study showed that the presence of a methoxy terminal group on PEG could elicit an immune response [45]. Thus, the nanoparticles prepared for this study had a methoxy end group on PEG, replaced in its synthesis process to attach a specific peptide for targeting. 

The current study extends the potential of the derivatized chitosan nanoparticles to deliver siRNA to an *in vivo* xenograft model of colon cancer. For which a peptide, CP15, was used that has shown its affinity towards SW480 colon cancer cells and not normal cells [28]. Other similar receptor-targeted nanoparticle studies include the use of human serum albumin (HSA) nanoparticles, tagged with trastuzumab antibody to deliver antisense oligonucleotides (ASOs) against PLK1 target [46, 47]. This study incorporates the use of an antibody as a targeting moiety for cell-specific delivery. The use of antibodies does add to the specificity of the targeting nanoparticles, however, it increases the size of the nanoparticles. In our study, we used a small peptide sequence to make the nanoparticle target specific because of their small size and ease of being used in organic solvents for conjugations. Another recent study uses liponanoparticles to deliver siRNA via intratumoral injections [48]. Lipid-based nanoparticles have been widely used in various therapeutic studies; however, their nonspecificity is a limitation when intended to deliver drugs/genes systemically. Thus, their use is mostly limited to local administration. Thus, the nanoparticle formulation developed in this study has an advantage of being able to customize by attaching a specific targeting ligand based on specific cell surface receptor. The incorporation of a peptide with its specific affinity towards SW480 cells was assumed to play an active targeting role for the nanoparticles. The tumor accumulation data as represented in Figure 3 suggests that the derivatized chitosan nanoparticles were able to target the tumor tissue as effectively as the unmodified chitosan nanoparticles, when compared with the siRNA delivered alone and the untreated control. Thus, it can be inferred that the systemic tumor targeting, could majorly be a size-dependent phenomenon due to the EPR effect rather than receptor-mediated targeting [36]. However, the biodistribution study as presented in Figure 4 showed that the CS-PEG-CP15 nanoparticles accumulated less in other organs, when compared to the unmodified chitosan nanoparticles (kidneys, *P* < 0.046). Thus, it can be inferred that the presence of a targeting moiety on the nanoparticles restricted their uptake by other tissues [49]. The enhanced circulation and lack of accumulation in other organs of CS-PEG-CP15 nanoparticles is attributed to the incorporation of PEG, which caters to the increased stability of the nanoparticles in blood, without being degraded or filtered by kidneys [50, 51]. However, since PEG does not have any charge, its uptake can become non-specific and can hinder endosomal escape and nucleic acid delivery, as also indicated by other studies [52]. Thus the incorporation of CP15 as a ligand/moiety was of an advantage to ensure the uptake by cancerous cells and not normal cells. It is noted that the cell staining in tissues receiving scrambled biotin-siRNA delivered without the carrier was not significant, as it is known that an siRNA delivered without a carrier is rapidly cleared from the system, within 15 min of its administration [53]. A recent study showed incorporation of a fusogenic peptide on the surface of a PEI graphed mesoporous silica nanoparticles for intratumoral siRNA delivery. The nanoparticles were tested on lung cancer and human cervical cancer cell lines. The results indicated successful knockdown of the VEGF gene by suppressing neovascularization of the tumors [54]. The use of the fusogenic peptide in the nanoparticle formulation was to aid the endocytic uptake of nanoparticles by the cells, followed by rapid endosomal escape [54]. Likewise, in our study, it is apparent that presence of the peptide facilitated the process of directing nanoparticles towards the cancer tissue in comparison to the unmodified chitosan nanoparticles. Moreover, the presence of PEG and CP15 peptide did not hinder the delivery of siRNA into the cancer cells, and the presence of available amines in chitosan polymer aided in rapid release of the siRNA from the endosome. This is confirmed by the staining of biotin tag that was specifically attached to the siRNA and not to the nanoparticles. Thus, just like PEI, the amines in chitosan present in the derivatized chitosan nanoparticles were able to cause endosomal escape and release siRNA in the cell's cytoplasm. Another similar study evaluates the ability of PEI graphed-PEGylated-RGD nanoparticles to systemically deliver pDNA to colon adenocarcinoma cell line in a mouse xenograft model [55]. Our study is in accordance with their findings, based on biodistribution study, that the presence of a peptide on the derivatized chitosan nanoparticles preferentially guided them towards the tumor tissue, with decreased accumulation to other organs as compared to the unmodified chitosan nanoparticles.

The efficacy study was performed with 6 animals in each group receiving different treatment formulations for 2 weeks. The formulations were delivered intraperitoneally to animals. The intraperitoneal dose administration was preferred to avoid the inconvenience caused by the intravenous injections to the animals. Since our previous *in vitro* studies have revealed the extreme cytotoxicity of unmodified chitosan nanoparticles, its use in the current *in vivo* study was not evaluated to ensure the anticancer effect was due to the therapeutic siRNA targeted against the PLK1 gene delivered by the nanoparticles and not by the nanoparticles themselves. The siRNA used against the PLK1 gene in this study, bears 2′-O-methyl modification. The 2′-O-methyl modification confers enhanced stability to siRNA against serum nucleases [56] and can also prevent type 1 interferon (IFN) induction and toll-like receptor (TLR) activation [57]. A detailed review of siRNA modifications for effective targeting and gene silencing is published elsewhere [58]. The RTPCR study performed on the harvested tumor tissues reveals 50.7 ± 19.5% gene silencing (*P* = 0.031) with PLK1 siRNA delivered by CS-PEG-CP15 chitosan nanoparticles as compared to the control (Figure 5), and PLK1 siRNA (*P* = 0.539) delivered alone had no effect on the PLK1 gene expression compared with the control. This indicates that the derivatized polymer had no toxic effect of its own and the PLK1-siRNA was solely responsible for gene silencing. The similar results were obtained in protein analysis (Figure 6), where the treatment group with PLK1-siRNA delivered by CS-PEG-CP15 nanoparticles showed significant suppression of PLK1 protein (*P* = 0.038) as compared with the untreated control. The serum safety markers were analyzed for liver function tests alanine ALT/AST, which provide an indication of possible hepatotoxicity. A test for CRP was also analyzed, which indicates systemic inflammation (Figure 7). Results showed no significant difference among different study groups, when compared with the control, thus, indicating no systemic toxicity posed by the CS-PEG-CP15-siRNA formulation when compared with the control group. 

This study suggests the potential application of peptide-tagged PEGylated nanoparticles for systemic siRNA delivery, targeting cancer. The current work stems from the development of a nanoparticle formulation that can be tailor-made for any specific cancer application by attaching the cell-specific peptide for targeting. This ability strengthens the use of nanoparticle based-delivery carriers as a platform to be used to deliver siRNAs, shRNAs, pDNAs, drugs, vaccines, proteins and peptides. The biodegradability, minimal toxicity and multifunctionality of these nanoparticles make their use favourable for biomedical applications. However, future work would still be needed to explore the full potential of these nanoparticles in terms of efficacy, tumor reduction, and so forth. Currently, studies are underway in our laboratory to carry an extensive *in vivo* study with prolonged treatment duration and increased animal number per group to see a therapeutic effect on the tumor tissue. Extra controls in the experiment will also be included, such as, other commercially available drug/gene delivery devices/transfection reagents, nontargeted nanoparticles to conclusively comment on the targeting ability of the developed nanoparticles.

## 5. Conclusion 

The current study projects the potential of synthesized peptide-tagged PEGylated chitosan nanoparticle formulation to be used *in vivo* in a mouse xenograft model of colorectal cancer. These nanoparticles prepared from a chemically modified polymer can be tailor-made by incorporation of a specific peptide which shows affinity towards a particular cell line. The nanoparticles, complexed siRNA at an N : P ratio of 129.2 and were >200 nm in size. The nanoparticles delivered the siRNA to the targeted site and caused 50.7 ± 19.5% suppression in the expression of PLK1, at mRNA level. These nanoparticles did not induce any immunological reactions and liver toxicity as determined by the serum analysis. This study shows a potential of using nanoparticles-mediated gene delivery that can be achieved via noninvasive strategy.
